# Role of Oxidative Stress and Autophagy in Thoracic Aortic Aneurysms

**DOI:** 10.1016/j.jacbts.2021.08.002

**Published:** 2021-10-25

**Authors:** Francesco G. Irace, Vittoria Cammisotto, Valentina Valenti, Maurizio Forte, Leonardo Schirone, Simona Bartimoccia, Alessandra Iaccarino, Mariangela Peruzzi, Sonia Schiavon, Andrea Morelli, Antonino G.M. Marullo, Fabio Miraldi, Cristina Nocella, Ruggero De Paulis, Umberto Benedetto, Ernesto Greco, Giuseppe Biondi-Zoccai, Sebastiano Sciarretta, Roberto Carnevale, Giacomo Frati

**Affiliations:** a Department of Cardiac Surgery, European Hospital, Rome, Italy; b Department of General and Specialized Surgery “Paride Stefanini,” Sapienza University of Rome, Rome, Italy; c Department of Clinical Internal, Anesthesiological, and Cardiovascular Sciences, Sapienza University of Rome, Rome, Italy; d Department of Medical-Surgical Sciences and Biotechnologies, Sapienza University of Rome, Latina, Italy; e IRCCS Neuromed, Pozzilli (IS), Italy; f Department of Cardiothoracic Surgery, Humanitas Clinical and Research Centre, IRCCS, Milan, Italy; g Mediterranea Cardiocentro, Naples, Italy; h Bristol Heart Institute, University of Bristol, Bristol, United Kingdom

**Keywords:** autophagy, endothelial dysfunction, oxidative stress, thoracic aortic aneurysm, ATG5, autophagy protein 5, HBA, hydrogen peroxide break-down activity, HRP, horseradish peroxidase, NADPH, nicotinamide adenine dinucleotide phosphate, NO, nitric oxide, PAGE, polyacrylamide gel electrophoresis, ROS, reactive oxygen species, SDS, sodium dodecyl sulfate, sNox2-dp, soluble Nox2-derived peptide, TAA, thoracic aortic aneurysms, VSMC, vascular smooth muscle cell

## Abstract

Thoracic aortic aneurysms (TAA) pathogenesis and progression include many mechanisms. The authors investigated the role of autophagy, oxidative stress, and endothelial dysfunction in 36 TAA patients and 23 control patients. Univariable and multivariable analyses were performed. TAA patients displayed higher oxidative stress and endothelial dysfunction then control patients. Autophagy in the TAA group was reduced. The association of oxidative stress and autophagy with aortic disease supports the role of these processes in TAA. The authors demonstrate a putative role of Nox2 and autophagy dysregulation in human TAA. These findings could pinpoint novel treatment targets to prevent or limit TAA progression.

## Highlights

• Because autophagy and Nox2 activation were identified as possible mechanisms for preservation of vessel integrity, they could be useful biomarkers to predict risk of aneurysm rupture by detecting the presence of a subclinical aneurysm or monitoring their growth.
• Biomarkers such as molecules involved in autophagic machinery or Nox2 activation may help to explain pathological processes involved in TAA development and expansion, thereby opening up novel potential therapeutic strategies, such as the use of natural activators of autophagy or molecules that inhibit Nox2 activation, in the setting of aneurysmatic pathology.
• Formation of aortic aneurysmal disease is multifactorial. Among the mechanisms involved, there is endothelial damage, oxidative stress, as well as an autophagy process, that seem to play a key role in TAA. Therefore, to identify the molecular mechanisms of these processes in TAA patients could lay the groundwork for defining strategies for preventing and slowing the progression of TAA.

## Visual Abstract

**Figure:**
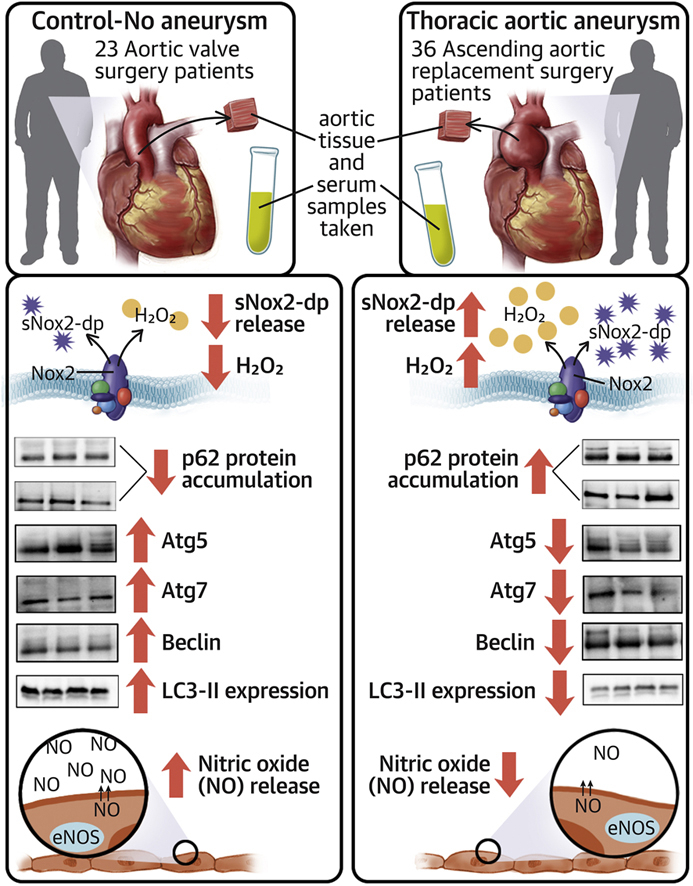

Aortic aneurysm is a segmental, full-thickness dilatation of the aorta, exceeding the normal vessel diameter by 50%, which represents the second most frequent aortic disease after atherosclerosis. Aortic aneurysm is often considered a “silent killer” due to its natural predisposition to dissection and/or sudden rupture (1). Formation of aortic aneurysmal disease is multifactorial and involves inflammation (2); infiltration of aortic wall by lymphocytes and macrophages (3); destruction of elastin and collagen in the media and adventitia by proteases, including matrix metalloproteinases (4, 5, 6); loss of smooth muscle cells with weakening of the media (7); and neovascularization (8). More recently, endothelial derangement, which is characterized by endothelial dysfunction and oxidative stress, has emerged as key contributor to thoracic aortic aneurysms (TAA). Nox2, which is the main isoform of nicotinamide adenine dinucleotide phosphate (NADPH) oxidase, and its role in the development of TAA still remains to be fully clarified.

Moreover, emerging data have recently suggested a possible role of autophagy in development and progression of aortic aneurysm. Autophagy is an intracellular process that is in charge for the degradation of senescent or dysfunctional cellular components through their sequestration by vesicles called autophagosomes and their subsequent degradation in lysosomes. Autophagy machinery is a complicated network of autophagy-related (ATG) proteins, which are involved in the induction and formation of autophagosomes. Autophagy is implicated in the regulation of endothelial homeostasis and function, also by limiting oxidative stress (9).

Few studies have investigated the possible association of autophagy with aortic aneurysm, with none specifically focused to date on TAA (10, 11, 12). Autophagy-related genes were found to be deregulated in human abdominal aneurysmatic tissues, but it remains unclear whether the autophagic process is inhibited or activated, particularly in TAA (13). Activation of autophagy is beneficial for cells in response to stress, including endothelial cells. Autophagy was found to preserve endothelial function through enhanced mitochondrial quality control, attenuation of endoplasmic reticulum stress, reactive oxygen species (ROS) production and inflammatory signaling, and increased nitric oxide (NO) bioavailability.

However, the interplay among autophagy, Nox2, oxidative stress, and TAA still remains to be elucidated. We hypothesized that oxidative-redox balance and autophagy are deranged in patients with TAA and are associated with TAA formation and progression.

## Methods

### Study design

We conducted our study using a case-control design. Specifically, we included 36 patients with TAA and an indication for elective surgical treatment. Twenty-three control patients were also included, using as the selection criterion aortic stenosis/regurgitation with indication for elective surgery in the absence of TAA. All performed procedures were in accordance with international guidelines. This study was approved by the Medical Ethics Committee of the European Hospital in Rome, and all the samples used in this study were obtained from the Department of Cardiothoracic Surgery of the European Hospital, after informed consent acquisition.

Inclusion criteria were: 1) indication for elective surgery for ascending aortic aneurysm or root aortic aneurysm and possible concomitant aortic valve repair or sparing, or aortic valve replacement; 2) ejection fraction >20%; and 3) age between 18 and 85 years. Exclusion criteria were 1) myocardial infarction in the previous 6 months; 2) chronic inflammatory disease or disease of the immune system; 3) malignancy in the last 3 years; 4) active infections or infective endocarditis; and 5) use of drugs that may modify/interfere with autophagy balance.

No patients had a personal or familiar history of hereditary diseases, such as Marfan syndrome and Loeys–Dietz syndrome. All patients with TAA underwent ascending aortic replacement surgery, and tissue samples were preserved in Falcon-Eppendorf at −80 °C until their processing. For each patient, plasma and serum samples were also collected and stored at −80 °C until use.

### Aortic specimens preparation and ex vivo experiments

Serum and plasma samples, obtained by centrifuging the blood at 300 × *g* for 10 minutes, were stored at −80 °C until the time of the analysis. Aortic tissue was immediately collected after surgical excision and stored at −80 °C. Subsequently, the tissue was homogenized, and supernatant collected for biochemical analysis.

### Serum and tissue sNox2-dp release

Nox2 activation was measured as soluble Nox2-derived peptide (sNox2-dp) with an enzyme-linked immunosorbent assay method as previously reported (14). Briefly, the peptide is recognized by binding to a specific monoclonal antibody against the amino acid sequence (224-268) of the external portion of Nox2, which was released following platelet activation. The enzyme activity is measured spectrophotometrically by the increased absorbency at 450 nm. Values were expressed as picograms per milliliter; intra-assay and interassay coefficients of variation were 8.95% and 9.01%, respectively.

### Serum and tissue nitric oxide bioavailability

In order to measure NO bioavailability in serum and in tissue lysates, we used a colorimetric assay kit (Cell Biolabs) that quantitatively measures NO by nitrite (NO^2−^)/nitrate (NO^3−^) determination. Briefly, the NO^3−^ in the sample is first converted to (NO^2−^ by nitrate reductase enzyme; next, total nitrite is detected with Griess Reagents as a colored dye product (absorbance 540 nm). Intra-assay and interassay coefficients of variation were <10%.

### Serum and tissue hydrogen peroxide determination

Hydrogen peroxide in serum and tissue lysates was evaluated by a Colorimetric Detection Kit (Arbor Assays) and expressed as μmol/L. In brief, samples are mixed with a colorless colorimetric substrate and the reaction initiated by addition of horseradish peroxidase (HRP). The reaction is incubated at room temperature for 15 minutes. The HRP reacts with the substrate in the presence of hydrogen peroxide to convert the colorless substrate into a pink-colored product. The pink product is read at 560 nm. Values were expressed as μmol/L. Intra-assay and interassay coefficients of variation were 2.1% and 3.7%, respectively.

### Serum hydrogen peroxide scavenging activity

The evaluation of the ability to detoxify hydrogen peroxide was assessed by the analysis of the hydrogen peroxide break-down activity (HBA) in serum with an HBA assay kit (Aurogene, code HPSA-50). The percentage of HBA was calculated according to the following formula: % of HBA = [(Ac − As) / Ac] × 100, where Ac is the absorbance of hydrogen peroxide 1.4 mg/mL, and As is the absorbance in the presence of the serum sample.

### Plasmatic P62 detection

Plasmatic P62 was analyzed with sandwich enzyme immunoassay technology (FineTest, No. EH10842). The concentration of protein can be calculated by the reading the O.D. absorbance at 450 nm. Values were expressed as nanograms per milliliter. Intra-assay and interassay coefficients of variation were <8% and <10%, respectively.

### Plasmatic autophagy protein 5 detection

For the quantitative determination of autophagy protein 5 (ATG5) concentrations in plasma samples, we used the sandwich enzyme immunoassay technique (FineTest, No. EH1729). The samples concentration was determined using a microplate reader set to 450 nm, and values were expressed as nanograms per milliliter. Intra-assay and interassay coefficients of variation were ≤8% and ≤12%, respectively.

### Protein extraction and Western blotting analysis

The human aortic tissue was placed in round-bottom microcentrifuge tubes and frozen at −80 °C for later homogenization. Briefly, 1) weigh a 5-mg piece of tissue and add 300 μL of ice-cold RIPA buffer (25 mmol/L Tris HCl [pH 7.6], 150 mmol/L NaCl, 1% NP-40, 1% sodium deoxycholate, 0.1% sodium dodecyl sulfate [SDS]) with protease and phosphatase inhibitors cocktail (Thermo Fisher Scientific) to preserve protein integrity and function after performing tissue lysis; 2) homogenize manually with a pestle to reduce tissue piece to very fine particles; 3) place in ice and maintain constant agitation for 1 hour at 4 °C; 4) centrifuge for 20 minutes at 12,000 rpm at 4 °C in a microcentrifuge. Gently remove the tubes from the centrifuge and place on ice, aspirate the supernatant, and then discard the pellet; and 5) briefly vortex tubes containing the supernatant previously collected, and proceed to sonication (3-5 sonication cycles 30 seconds each, at 4 °C).

The protein concentration was determined by Bradford assay (Bio-Rad Laboratories). Equal amounts of protein (30 μg/lane) were solubilized in a 2× Laemmli sample buffer containing 20% of 2-mercaptoethanol and were electrophoretically separated on a 15% SDS-polyacrylamide gel electrophoresis (PAGE) and then electrotransferred to nitrocellulose membranes. After blocking with bovine serum albumin (5%, Sigma Aldrich), the membranes were incubated with monoclonal anti-LC3 antibody (MBL International), or anti-ATG5 antibody (Novus Biological) or anti-ATG7 antibody (Cell Signaling Technology) or anti-Beclin antibody (Cell Signaling Technology) and monoclonal anti-βactin antibody (Santa Cruz Biotechnology), and incubated at 4 °C overnight. Finally, membranes were incubated with the secondary antibody (Santa Cruz Biotechnology, 1:5,000). The densitometry analysis was performed using ImageLab software 6.1.

### Soluble/insoluble fractionation protocol

Tissue samples were cut into fragments of 30 mg weight and homogenized in 250 μL of an ice-cold enzyme digestion mix solution (soluble lysis buffer, 10 mmol/L TRIS [pH 7.4], 1% Triton-X 100, 150 mmol/L NaCl, 10% glycerol), added with protease and phosphatase inhibitors cocktail (10 μg/mL, Thermo Fisher Scientific) and 20 mmol/L *N*-ethylmaleimide (Sigma Aldrich), and solubilized in an ethanolic solution of phenylmethysulfonyl fluoride 100 mmol/L (ChemCruz). Samples were kept on ice for 1 hour, then centrifuged at 15,000 × *g* for 20 minutes at 4 °C, and the supernatant was collected as the “soluble” fraction.

Pellet was washed 2 times with 500 μL of soluble lysis buffer. After each wash, samples were centrifuged at 15,000 × *g* for 5 minutes at 4 °C, and the supernatant was removed. Pellets were then resuspended with 150 μL of insoluble lysis buffer (10 mmol/L TRIS [pH 7.4], 1% Triton-X 100, 150 mmol/L NaCl, 10% glycerol, and 4% SDS). Samples were sonicated for 30 seconds at room temperature with a probe sonicator (125 W, 20 kHz pulse, amplitude 40%), then boiled for 30 minutes and briefly centrifuged (1 minute at 6,000 × *g*) to collect the entire sample. Protein concentration was determined by detergent compatible protein assay (Bio-Rad Laboratories). Equal amounts of protein (30 μg/lane) were solubilized in a 2× Laemmli sample buffer containing 20% 2-mercaptoethanol. Soluble fraction proteins were resolved by 12% SDS-PAGE, whereas insoluble fraction proteins were resolved by 8% SDS-PAGE and transferred onto a 0.45 μmol/L nitrocellulose membrane. After blocking with 5% bovine serum albumin, membranes were incubated overnight at 4 °C with p62 antibody (Abnova) (1:1,000) or monoclonal anti-βactin antibody (Santa Cruz Biotechnology) (1:1,000). Membranes were then incubated for 1 hour at room temperature with HRP-conjugated secondary antibody (1:3,000, Santa Cruz Biotechnology) and then detected by enhanced chemiluminescence. Densitometric analysis of bands was performed using ImageLab software. Results were expressed as arbitrary units.

### RNA extraction and real-time polymerase chain reaction

Frozen aorta samples (30-60 mg) were lysed in 700 μL of ice-cold Qiazol and homogenized using Ultra-turrax (IKA). Total RNA was extracted using the miRNeasy Mini Kit (Qiagen), and quantified using a spectrophotometer. cDNA was synthesized using 0.5 μg of RNA, with the High-Capacity cDNA Reverse Transcription Kit (Life Technologies, Thermo Fisher Scientific). Real-time quantitative polymerase chain reaction was performed on a 7900HT thermocycler to assess gene expression, using Power SYBR Green PCR Master Mix (Life Technologies, Thermo Fisher Scientific) and standard thermocycling conditions according to the manufacturer’s protocol.

The relative expression of each gene was calculated using the comparative Ct method (2^−ΔΔCt^) for each patient sample compared with a control sample. GAPDH was selected as the housekeeping gene according to the Applied Biosystems Software housekeeping gene stability score calculation. The set of genes analyzed and the primers sequences are listed in Supplemental Table 1.

### Statistical analysis

Continuous variables are reported as median with 25th and 75th percentiles (interquartile range), and categorical variables as count with percentage. Distribution of continuous variables was first inspected using histograms and then compared between groups using nonparametric Mann-Whitney *U* test. Categorical variables were compared with Fisher's exact test. Correlation analysis was performed with using Spearman's rho test. For exploratory purposes, we appraised the impact of potential moderators of oxidation and autophagy features with a series of univariable and multivariable linear regression analyses, confirming main modelling results with nonlinear models. No formal sample size analysis was performed, because enrollment was based on a representative 2-year caseload at participating centers. Statistical significance was set at 2-tailed test *P* < 0.050 level, without multiplicity adjustment. Statistical analysis was performed with Prism 7 software (GraphPad Software) and Stata 13 software (StataCorp).

## Results

### Patients

From February 2019 to June 2021, we enrolled 59 patients, who were assigned to 2 groups according to their pathology and accomplished surgery: 1) 36 TAA patients; and 2) 23 aortic valve surgery patients (control patients). Baseline characteristics are summarized in Table 1. Notably, there were no significant differences between the 2 groups, except for height (171 [165-180] cm vs 164 [158-178] cm; *P* = 0.016), body surface area (1.94 [1.77-2.07] m^2^ vs 1.76 [1.72-2.01] m^2^; *P* = 0.044), and dyslipidemia (16 [44.4%] vs 18 [78.3%]; *P* = 0.015). Echocardiographic analysis showed that patients with TAA had larger left ventricular diameters (52 [47-57] mm vs 42 [40-48] mm; *P* ≤ 0.001) and thickness (11 [11-13] mm vs 13 [12-14] mm; *P* = 0.003), as well as aortic diameters (51 [50-53] mm vs 35 [32-39] mm; *P* < 0.001) (Table 2). Similarly, severe aortic stenosis was less common in the TAA group (5 [13.9%] vs 19 [82.6%]; *P* < 0.001). Laboratory analysis showed similar features in the 2 groups, whereas statin therapy appeared less common in the TAA group (8 [22.2%] vs 13 [56.5%]; *P* = 0.012) (Supplemental Table 2).

**Table 1:** Baseline Patient Characteristics

	TAA Group (n = 36)	Control Group (Aortic Valve Surgery) (n = 23)	*P* Value
Age, y	65 (56-72)	70 (64-73)	0.13
Male	28 (77.8)	12 (52.2)	0.050
Height, cm	171 (165-180)	164 (158-178)	0.016
Weight, kg	82 (69-89)	75 (66-83)	0.21
BSA, m^2^	1.94 (1.77-2.07)	1.76 (1.72-2.01)	0.044
BMI, kg/m^2^	25.9 (25.2-28.4)	26.9 (24.5-28.1)	0.84
Family history of CVD	9 (25.0)	5 (21.7)	1.00
Tobacco use			0.80
Active	9 (25.0)	4 (17.4)	
Former	5 (13.9)	4 (17.4)	
Ever	22 (61.1)	15 (65.2)	
Liver disease	0	1 (4.4)	0.39
Alcoholism	0	1 (4.4)	0.39
Hypertension	28 (77.8)	20 (87.0)	0.50
Diabetes mellitus	1 (2.8)	3 (13.0)	0.29
Dyslipidemia	16 (44.4)	18 (78.3)	0.015
COPD	4 (11.1)	1 (4.4)	0.64
Obesity	2 (5.6)	0	0.52
Atrial fibrillation			0.30
Permanent	1 (2.8)	3 (13.0)	
Paroxysmal	4 (11.1)	1 (4.4)	
Ever	31 (86.1)	19 (82.6)	
Prior PM implantation	0	1 (4.4)	0.39
Prior AMI	0	0	1.00
Prior PCI	0	2 (8.7)	0.15
Prior CVA	0	1 (4.4)	0.39
Heart failure	1 (2.8)	0	1.00

Values are median (interquartile range) or n (%).

AMI = acute myocardial infarction; BMI = body mass index; BSA = body surface area; COPD = chronic obstructive pulmonary disease; CVA = cerebrovascular accident; CVD = cardiovascular disease; PCI = percutaneous coronary intervention; PM = pacemaker; TAA = thoracic aortic aneurysms.

**Table 2:** Echocardiographic Characteristics

	TAA Group (n = 36)	Control Group (Aortic Valve Surgery) (n = 23)	*P* Value
Heart rate, beats/min	61 (57-67)	66 (60-71)	0.28
LVEF, %	60 (56-65)	60 (57-63)	0.81
LVEDD, mm	52 (47-57)	42 (40-48)	<0.001
LVESD, mm	35 (32-40)	29 (27-31)	<0.001
IVS, mm	11 (11-13)	13 (12-14)	0.003
PW, mm	11 (11-12)	12 (11-13)	0.055
Aortic diameters, mm			
TT	50 (40-52)	36 (32-39)	<0.001
STJ	36 (34-42)	30 (26-32)	<0.001
VS	43 (39-50)	34 (30-36)	<0.001
Aortic annulus	25 (23-26)	21 (20-21)	<0.001
Maximum diameter	51 (50-53)	35 (32-39)	<0.001
TAPSE, mm	22 (21-27)	22 (19-24)	0.17
Bicuspid aortic valve	8 (22.2)	6 (26.1)	0.76
Severe aortic stenosis	5 (13.9)	19 (82.6)	<0.001
Aortic regurgitation			0.21
None	8 (22.2)	7 (30.4)	
Trivial	8 (22.2)	5 (21.7)	
Mild	4 (11.1)	6 (26.1)	
Moderate	9 (25.0)	1 (4.4)	
Severe	7 (19.4)	4 (17.4)	
Mitral regurgitation			0.20
None	12 (33.3)	5 (21.7)	
Trivial	18 (50.0)	13 (56.5)	
Mild	6 (16.7)	2 (8.7)	
Moderate	0	2 (8.7)	
Severe	0	1 (4.4)	
Tricuspid regurgitation			0.54
None	17 (47.2)	8 (34.8)	
Trivial	17 (47.2)	12 (52.2)	
Mild	2 (5.6)	2 (8.7)	
Moderate	0	1 (4.4)	
Severe	0	0	
Diastolic dysfunction			0.088
None	21 (58.3)	8 (34.8)	
Trivial	11 (30.6)	14 (60.9)	
Mild	3 (8.3)	1 (4.4)	
Moderate	1 (2.8)	1 (4.4)	
Severe	0	0	
Systolic pulmonary artery pressure, mm Hg	27 (22-31)	28 (20-33)	0.64

Values are median (interquartile range) or n (%).

IVS = interventricular septum; LVEDD = left ventricular end-diastolic diameter; LVEF = left ventricular ejection fraction; LVESD = left ventricular end-systolic diameter; PW = posterior wall; STJ = sinotubular junction; TAA = thoracic aortic aneurysms; TAPSE = tricuspid annulus plane systolic excursion; TT = tubular tract; VS = Valsalva sinuses.

Moreover, the 2 groups did not show any significant differences in laboratory findings at admission routine exam, except for basophil counts and erythrocyte sedimentation rate, coronary angiography findings, and admission pharmacological therapy; these data are available in Supplemental Table 1.

### Oxidative stress and antioxidant system in TAA patients and control patients

**Figure 1:**
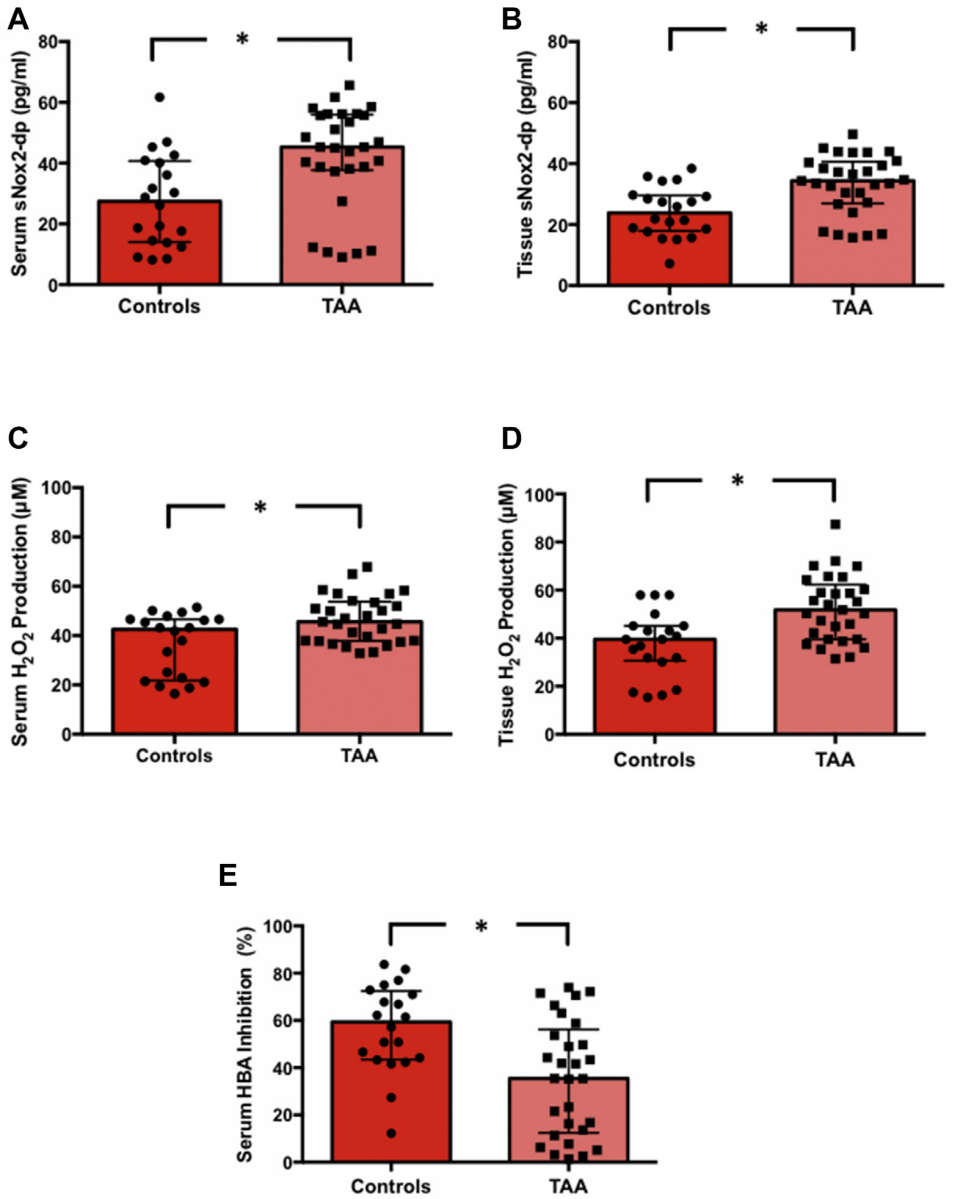
Systemic and Tissue Oxidative Stress in TAA Serum **(A)** and tissue **(B)** soluble Nox2-derived peptide (sNox2-dp) release, serum **(C)** and tissue **(D)** hydrogen peroxide production, and serum hydrogen peroxide break-down activity (HBA) **(E)**, in the control group (n = 20) and the thoracic aortic aneurysms (TAA) group (n = 29). Data are expressed as median and interquartile range. ∗*P* < 0.05.

Patients undergoing surgery for TAA showed higher levels of oxidative stress parameters, as compared with the control group. The TAA group showed higher levels of sNox2-dp in both serum (45.29 [38.07-55.77] pg/mL vs 27.43 [14.17-40.49] pg/ml; *P* = 0.005) and tissue samples (34.36 [27.23-40.37] vs 23.84 [18.11-29.49]; *P* = 0.003), with a positive and significantly different serum/tissue ratio (1.22 [1.15-1.29] vs 1.03 [0.76-1.18]; *P* = 0.015) (Supplemental Table 3, Figures 1A and 1B). Accordingly, patients with TAA had higher levels of hydrogen peroxide production in both serum (45.61 [37.94-53.44] μmol/L vs 42.53 [22.18-46.65] μmol/L; *P* = 0.020) and tissue samples (51.77 [39.50-64.31] vs 39.50 [33.65-45.15]; *P* = 0.003) (Supplemental Table 3, Figures 1C and 1D). Furthermore, we found a significantly lower ability to neutralize hydrogen peroxide in the TAA group using the HBA assay (35.42 [13.64-53.67] vs 59.35 [43.73-71.93]; *P* = 0.003) (Supplemental Table 3, Figure 1E).

### Autophagy in TAA patients and control patients

**Figure 2:**
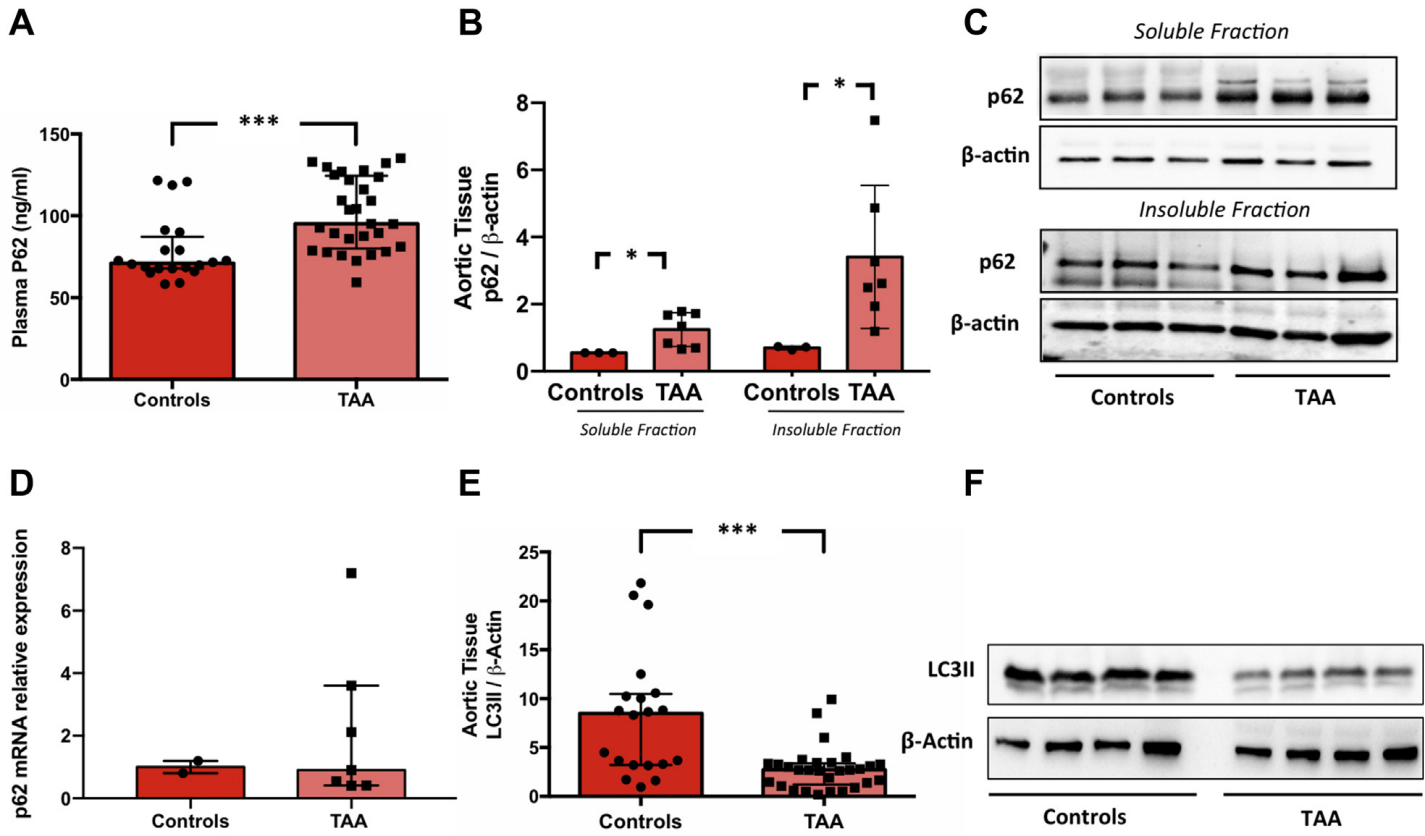
Role of Autophagy in TAA **(A)** Plasma p62 levels in the control group (n = 20) and the thoracic aortic aneurysms (TAA) group (n = 29). Quantification of total soluble and insoluble p62 fractions **(B)** and relative representative Western blot bands **(C)** in the control group (n = 3) and the TAA group (n = 7). Expression levels of p62 genes **(D)** in the control group (n = 2) and the TAA group (n = 7). LC3-II protein expression **(E)** and relative representative Western blot bands **(F)** in the control group (n = 20) and the TAA group (n = 29). ∗*P* < 0.05; ∗∗∗*P* < 0.001.

To investigate the role of autophagy, we compared the plasma levels of P62 in the TAA and control groups. In comparison to the control group, TAA group showed a significant increase of P62 levels (95.06 [81.11-123.67] ng/mL vs 70.98 [67.71-84.46] ng/mL; *P* < 0.001) (Supplemental Table 3, Figure 2A). To examine whether the solubility of p62 was altered in TAA group, cellular lysates were fractionated into Triton X-100–soluble and -insoluble fractions. The quantitative analysis showed that p62 was significantly increased in TAA as compared with the control group. Intriguingly, the quantitative analysis of the insoluble protein fractions revealed a prominent accumulation of p62 in TAA as compared with the control, suggesting the interruption of autophagic flux (Figures 2B and 2C). Moreover, we quantified the gene expression of p62 in tissue obtained from TAA patients and control patients. The results showed no significant differences in p62 mRNA expression in TAA tissue as compared with control (Figure 2D). Finally, we evaluated autophagy by investigating the expression of LC3-II with Western blot analyses revealing that the expression of LC3-II protein was significantly reduced in the TAA group as compared with the control group (2.71 [1.38-3.34] vs 8.49 [3.25-10.40]; *P* < 0.001) (Supplemental Table 3, Figures 2E and 2F).

**Figure 3:**
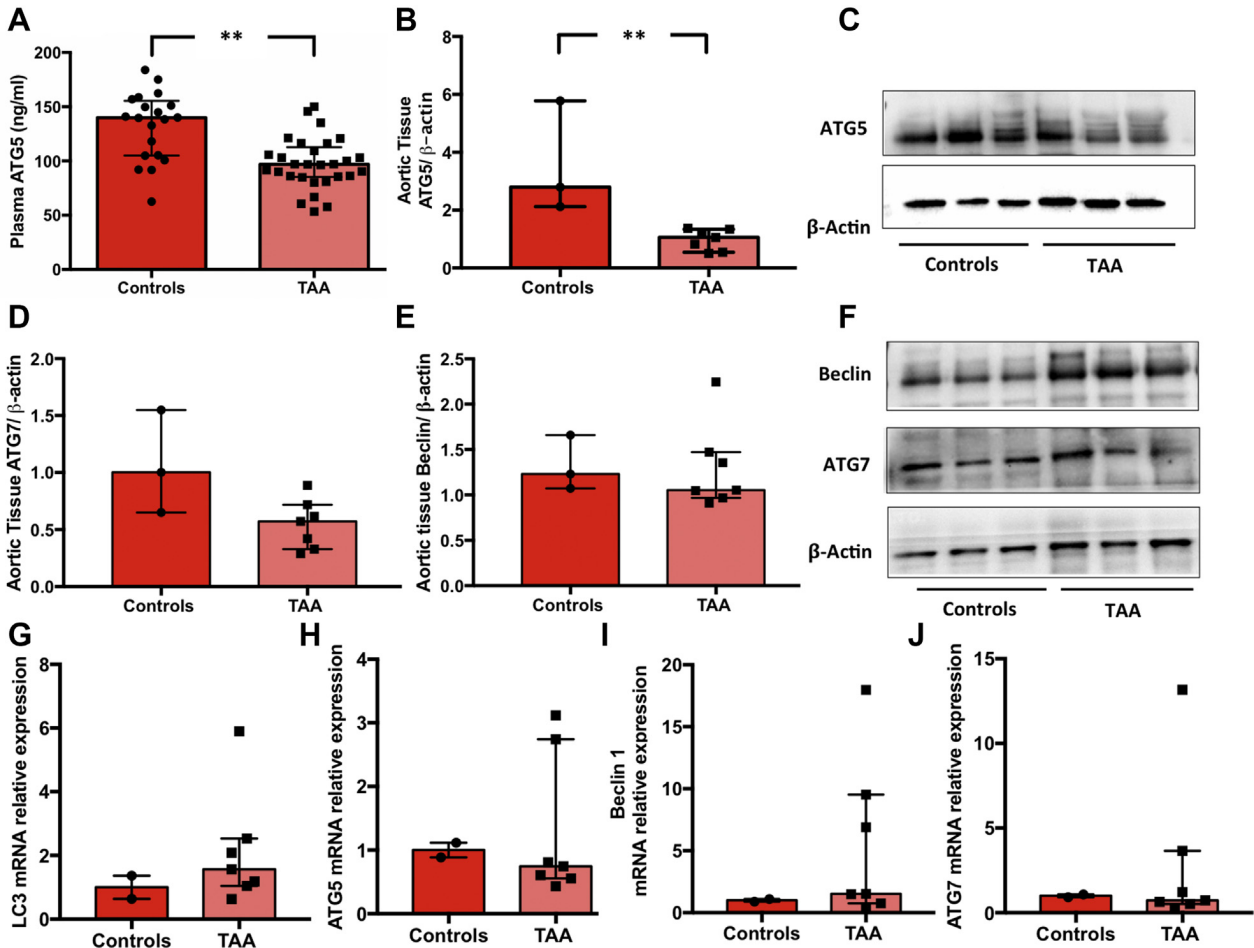
Role of Autophagy in TAA **(A)** Plasma ATG5 levels in the control group (n = 20) and the thoracic aortic aneurysms (TAA) group (n = 29). ATG5 **(B)**, ATG7 **(D)**, and Beclin-1 **(E)** protein expression and relative representative Western blot bands **(C and F)** in the control group (n = 3) and the TAA group (n = 7). Expression levels of autophagy-related genes LC3, ATG5, Beclin-1, and ATG7 **(G-J)** in the control group (n = 2) and the TAA group (n = 7). Data are expressed as median and interquartile range. ∗∗*P* < 0.01.

To further explore the autophagic flux, we examined other ATG proteins. Results showed in the same TAA group a significant reduction of ATG5 plasma levels (*P* = 0.003) (Supplemental Table 3, Figure 3A). Western blot analyses corroborated these data by showing a significant reduction of ATG5 (1.06 [0.55-1.34] vs 2.80 [2.12-5.78]; *P* = 0.017) and a trend toward a significant reduction in ATG7 expression (0.57 [0.33-0.72] vs 1.00 [0.65-1.55]; *P* = 0.053) in TAA samples, as compared with control samples (Figures 3B-3D and 3F). However, no significant changes in Beclin-1 levels were observed (1.18 [0.97-1.40] vs 1.44 [1.29-2.01]; *P* = 0.21) (Figures 3E and 3F). On the other hand, mRNA expression of LC3, ATG5, Beclin-1, and ATG7 tended to increase in aneurysm samples, although in a nonsignificant manner (Figures 3G-3J). These results indicate that transcriptional mechanisms do not contribute to autophagy inhibition, whereas post-translational mechanisms are likely to be involved (15,16).

### Endothelial dysfunction in TAA patients and control patients

**Figure 4:**
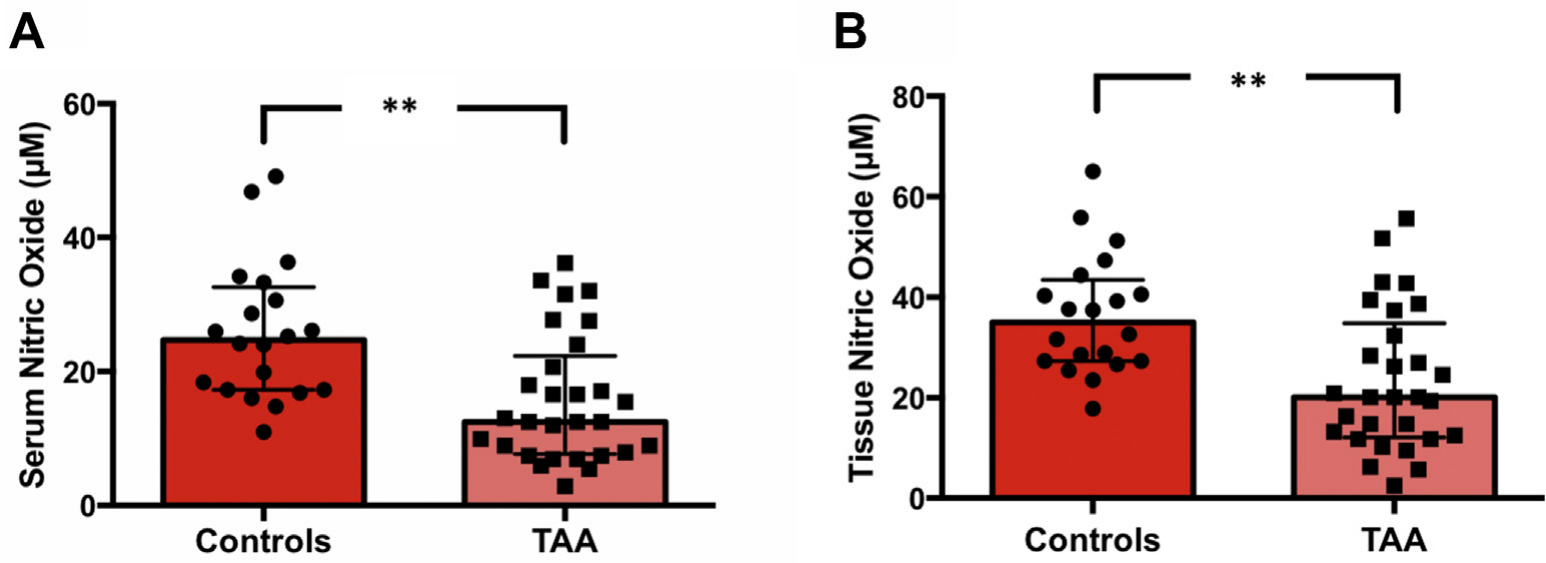
Systemic and Tissue Endothelial Function in TAA Serum **(A)** and tissue **(B)** NOx bioavailability in the control group (n = 20) and the thoracic aortic aneurysms (TAA) group (n = 29) as median and interquartile range. ∗∗*P* < 0.01.

For the evaluation of endothelial dysfunction, we measured the bioavailability of NO. The TAA group showed lower levels of NO in both serum (12.49 [7.92-20.66] μmol/L vs 24.72 [17.28-31.94] μmol/L; *P* = 0.001) and tissue samples (20.11 [12.50-32.36] μmol/L vs 35.04 [27.29-42.49] μmol/L; *P* = 0.001), with significantly different serum/tissue gradients as well (0.623 [0.605-0.645] vs 0.644 [0.633-0.650]; *P* = 0.010) (Supplemental Table 3, Figure 4).

### Correlation analysis

Results of correlation analysis are reported in Supplemental Table 4. Specifically, nonparametric correlation analysis showed several significant associations between sNox2 and tissue Nox2 (rho = 0.761; *P* < 0.001), serum NO (rho = −0.504; *P* < 0.001), tissue NO (rho = −0.453; *P* = 0.001), P62 (rho = 0.424; *P* = 0.004), and ATG5 (rho = −0.408; *P* = 0.004). Furthermore, tissue Nox2 was significantly associated with serum NO (rho = −0.480; *P* < 0.001), tissue NO (rho=-0.407; *P* = 0.004), P62 (rho=0.380; *P* = 0.007) and ATG5 (rho = −0.341; *P* = 0.016), whereas HBA was significantly associated with tissue hydrogen peroxide (rho = −0.295; *P* = 0.040). Serum hydrogen peroxide was significantly associated with tissue hydrogen peroxide (rho = 0.650; *P* < 0.001) and P62 (rho = 0.419; *P* = 0.003), whereas tissue hydrogen peroxide was significantly associated with P62 (rho = 0.525; *P* < 0.001). Serum NO was significantly associated with tissue NO (rho = 0.944; *P* < 0.001), P62 (rho = −0.293; *P* = 0.041), ATG5 (rho = 0.480; *P* < 0.001), and LC3/Actin (rho = 0.291; *P* = 0.042). Tissue NO was significantly associated with P62 (rho = −0.297; *P* = 0.038), ATG5 (rho = 0.482; *P* < 0.001), and LC3/Actin (rho = 0.376; *P* = 0.008). P62 was significantly associated with ATG5 (rho = −0.310; *P* = 0.030), and LC3/Actin (rho = −0.456; *P* = 0.001). Overall, these findings indicate that increased Nox2 activity and oxidative stress are associated with autophagy inhibition.

### Multivariable analysis

Multivariable analysis was performed to explore for potential moderators of oxidation and autophagy features, confirming the significant and independent association between group (TAA vs control) assignment and sNox2, tissue Nox2, hydrogen peroxide, serum NO, tissue NO, as well as LC3/Actin, even adjusting for clinical, laboratory, and imaging features (Supplemental Table 5).

## Discussion

The present study provides the first evidence to our knowledge that patients with TAA have decreased autophagy. This molecular derangement was associated with increased oxidative stress and activation of Nox2, a major source of oxidative stress in the vasculature.

Many vascular diseases are characterized by increased production of ROS in the vessel wall, which leads to reduction NO bioavailability and endothelial dysfunction (17). Moreover, Nox2 activation was also found to be involved in endothelial dysfunction and vascular damage in response to hypertension, diabetes, and aging (18, 19, 20). Nox2-derived ROS induce increased vascular expression of cytokines and adhesion molecules, which lead to recruitment of immune cells, vascular inflammation, fibrosis, remodeling and damage. Impaired NO bioavailability due to endothelial dysfunction and NADPH oxidase overexpression also directly contribute to vascular smooth muscle cell growth, proliferation, contraction, and differentiation, which are also major determinants of aneurysm development (21).

Our data further extend this notion by demonstrating the existence of a persistent increase of oxidative status along with reduced scavenging activity in TAA patients with respect to control patients. We observed higher levels of Nox2 activity and hydrogen peroxide, as well as a decline of serum HBA, both systemically and in aortic tissue. In addition, NO bioavailability was significantly reduced in TAA patients as compared with control patients.

Interestingly, we found that increased Nox2 activity and oxidative stress were associated with autophagy inhibition, confirming previous evidence demonstrating that oxidative stress inhibits autophagy (22). Autophagy is an intracellular degradation mechanism playing a paramount adaptive cellular modification in response to oxidative stress, characterized by elimination of excessive cellular ROS and maintenance of an appropriate redox balance. Autophagy deficiency leads to intracellular accumulation of dysfunctional mitochondria and misfolded proteins with a consequent increase of ROS generation (23). Previous works identified a key role for the autophagic machinery in regulating vascular smooth muscle cell (VSMC) death and endoplasmic reticulum stress–dependent inflammation with important impact on aortic wall homeostasis and repair. In particular, Clément et al (10) identified a critical role for ATG5 deficiency in VSMCs, which was able to hamper autophagosome formation, enhancing cell death and promoting the development of aortic dissection. In addition, ATG7 gene deletion in VSMCs was also found to inhibit autophagy and favor atherosclerosis and aortic aneurysm induced by high cholesterol levels, with subsequent reduced survival because of aortic rupture (11). More recently, a study revealed that FoxO3a promotes VSMC phenotypic switching to accelerate abdominal aortic aneurysm formation through the P62/LC3BII autophagy signaling pathway (24). Moreover, p62, IL-6, Rab7, and Atg5/IRE1α pathways of autophagy may play a role in aortic aneurysm and aortic dissection, and can be considered a novel super-selective therapeutic target (25).

Our study translated these experimental results to the clinical context demonstrating an abnormal expression of systemic markers of autophagy in TAA patients. Interestingly, we also found a significant reduction of protein expression of LC3, ATG5, and ATG7, important markers of autophagy in TAA samples, whereas levels of p62, a protein degraded by autophagy, were increased. These results indicate autophagy inhibition in aortic aneurysm samples. By contrast, mRNA levels of the same autophagy markers tended to be increased in aneurysm samples, likely as a compensatory mechanism to the impairment of the process. These results indicate that transcriptional mechanisms are not responsible for autophagy inhibition in aortic aneurysms, whereas post-translational modifications may play a major role. In recent years, post-translational modifications have emerged as critical mechanisms regulating autophagy in response to the actions of multiple intracellular signaling pathways (15,16). Basically, all autophagy-related proteins were found to undergo phosphorylation, acetylation, ubiquitination, O-GlcNAcylation, and redox modifications, which markedly affect their function and levels. In addition, it is known that post-translational modifications significantly affect protein stability and aggregation. Future studies are warranted to investigate the exact molecular mechanisms leading to autophagy in aneurysm samples.

The results of our study are potentially relevant. We identified a possible role for autophagy in the preservation of vessel integrity in human subjects. Autophagy and Nox2 activation could represent predictive markers in the setting of development and progression of TAA. Indeed, they could be useful biomarkers to detect the presence of a subclinical aneurysm or represent a valuable tool to monitor their expansion rate, thus predicting the risk of rupture.

Lastly, natural activators of autophagy are under investigation in the setting of several cardiovascular diseases with results of paramount importance (22,26,27). Interestingly, a recent work demonstrated that oral spermidine supplementation increases plasma spermidine levels and suppresses the development of experimental abdominal aortic aneurysms with preservation of aortic structural integrity by reducing inflammatory cell infiltration and circulating inflammatory monocytes, as well as increasing autophagy-related protein (28). These results suggest that natural activators of autophagy might be potentially useful for the treatment of human aortic aneurysms.

### Study limitations

This work has many drawbacks, including the observational features, the small sample size without a pre-hoc sample size/power analysis, and the fact that we cannot exclude the impact of other mechanisms intertwined with the inflammatory process in TAA (29). Furthermore, an animal modelling study is required for more explicit mechanistic proof of our hypothesis. Autophagy flux could not be assessed by using lysosome inhibitors because of the technical difficulties in performing such experiments with human samples. The exact molecular mechanisms leading to autophagy inhibition in TAA were not identified. Accordingly, further studies will be necessary to establish whether other mechanisms could contribute to development and progression of TAA. Finally, the small sample size and lack of multiple comparison adjustment should be noted, so results should be interpreted with caution, given the lack of control for type I error.

## Conclusions

Biomarkers such as molecules involved in autophagic machinery or Nox2 activation may help to explain pathological processes involved in TAA development and expansion, thereby opening up novel potential therapeutic strategies in the setting of aneurysmatic pathology.

Perspectives **COMPETENCY IN MEDICAL KNOWLEDGE:** Formation of aortic aneurysmal disease is multifactorial. Among mechanisms involved, there is endothelial damage, characterized by endothelial dysfunction and oxidative stress, as well as autophagy process, that seem to play a key role in TAA. Therefore, to identify the molecular mechanisms of these processes in TAA patients could lay the groundwork for defining strategies for preventing and slowing the progression of TAA. Because autophagy and Nox2 activation were identified as possible mechanisms for preservation of vessel integrity, they could represent predictive markers in the setting of development and progression of TAA. In particular, they could be useful biomarkers to predict risk of aneurysm rupture by detecting the presence of a subclinical aneurysm or monitoring their growth.

**TRANSLATIONAL OUTLOOK:** Additional research is needed to investigate the use of natural activators of autophagy or molecules that inhibit Nox2 activation that may be potentially useful for the treatment of human aortic aneurysms.

## Funding Support and Author Disclosures

This study was funded by Sapienza University to Prof Frati (Prot. RM11715C7852D47E). Prof. Biondi-Zoccai has consulted for Cardionovum, Crannmedical, Innovheart, Opsens Medical, Meditrial, and Replycare. All other authors have reported that they have no relationships relevant to the contents of this paper to disclose.
