# Supplementary material for: Role of Oxidative Stress and Autophagy in Thoracic Aortic Aneurysms
Source: JACC Basic Transl Sci. 2021 Oct 25;6(9-10):719–30. doi: 10.1016/j.jacbts.2021.08.002 (PMC8559314; doi:10.1016/j.jacbts.2021.08.002)
Supplement: Supplemental Tables 1–5 [file mmc1.pdf]

Supplemental Table 1. Genes analyzed and the primer sequences used for PCR

OLIGO name	Sequence 5'-3'
ATG5_FW	AGAAGCTGTTTCGTCCTGTGG
ATG5_REV	AGGTGTTTCCAACATTGGCTC
ATG7_FW	ATGATCCCTGTAACTTAGCCCA
ATG7_REV	CACGGAAGCAAACAACTTCAAC
BECLIN1_FW	TGGGGAGGTTAGGATTTGGGA
BECLIN1_REV	GAGCCGTAGGGTGGAAAGC
LC3B_FW	CCGCACCTTCGAACAAAGAG
LC3B_Rev	AAGCTGCTTCTCACCCTTGT
SQSTM1(P62)_FW	AAATGGGTCCACCAGGAAAC
SQSTM1(P62)_REV	TTCAGGAAATTCACACTCGGA
q-GAPDH_FW	ATCAGCAATGCCTCCTGCAC
q-GAPDH_REV	TGGCATGGACTGTGGTCATG

Supplemental Table 2. Additional features.*

Feature	TAA Group (N=36)	Control Group (Aortic Valve Surgery) (N=23)	P
Hb – g/dl	14.3 (13.5, 15.2)	14.2 (12.6, 15.1)	0.26
**RBC – 10^6/ml**	4.73 (4.36, 5.05)	4.70 (4.26, 4.92)	0.38
**Hct – %**	42.1 (39.7, 44.9)	41.6 (38.2, 45.2)	0.25
**WBC – 10^3/ml**	7.55 (6.70, 8.65)	7.10 (6.30, 8.60)	0.47
**Neutrophils – %**	60.9 (54.3, 64.1)	58.4 (50.9, 68.4)	0.99
**Eosinophils – %**	2.9 (1.7, 3.5)	2.4 (1.7, 3.0)	0.35
**Basophils – %**	0.2 (0.1, 0.3)	0.1 (0.1, 0.2)	0.018
**Lymphocytes – %**	29.3 (27.2, 36.9)	31.4 (25.7, 39.6)	0.74
**Monocytes – %**	6.1 (5.0, 7.2)	5.4 (4.4, 7.1)	0.21
**PLT – 10^3/ml**	210 (181, 256)	203 (186, 252)	0.61
**Creatinine – mg/dl**	0.94 (0.84, 1.05)	0.95 (0.84, 1.09)	0.98
**BUN – mg/dl**	42 (38, 55)	41 (38, 55)	0.79
**Glycemia – mg/dl**	104 (100, 111)	102 (86, 119)	0.68
**Cholesterol – mg/dl**	181 (145, 205)	181 (143, 219)	0.93
**LDL – mg/dl**	108 (90, 120)	106 (91, 130)	0.66
**HDL – mg/dl**	49 (40, 69)	52 (41, 60)	0.76
**Triglycerides mg/dl**	152 (87, 215)	125 (94, 144)	0.18
**ALT – U/l**	19 (15, 24)	16 (11, 24)	0.33
**AST – U/l**	19 (16, 23)	19 (15, 23)	0.96
**CRP – mg/dl**	0.22 (0.09, 0.35)	0.12 (0.06, 0.20)	0.14
**ESR – mm/h**	3 (2, 7)	7 (3, 18)	0.018
**Coronary angiography findings – no. (%)**			0.32
**No coronary arteries disease**	30 (83.3)	15 (65.2)	
**One-vessel disease**	1 (2.8)	3 (13.0)	
**Two-vessels disease**	3 (8.3)	3 (13.0)	
**Three-vessel disease**	2 (5.6)	2 (8.7)	
**ASA – no. (%)**	8 (22.2)	10 (43.5)	0.15
**BB – no. (%)**	16 (44.4)	12 (52.2)	0.60
**ACEi – no. (%)**	12 (33.3)	8 (34.8)	1
**ARB – no. (%)**	8 (22.2)	8 (34.8)	0.37
**CCB – no. (%)**	7 (19.4)	3 (13.0)	0.73
**Diuretics – no. (%)**	10 (27.8)	8 (34.8)	0.58
**Statins – no. (%)**	8 (22.2)	13 (56.5)	0.012
**PPI – no. (%)**	19 (52.8)	17 (73.9)	0.17
**Levothyroxine – no. (%)**	1 (2.8)	4 (17.4)	0.070

*presented as median with 25th and 75th percentiles (Q1, Q3) and corresponding p value, or as count with percentage (%) with corresponding p value

ALT: alanine aminotransferase, ASR: aspartate aminotransferase, BUN: blood urea nitrogen, CRP: C-reactive protein, ESR: erythrocyte sedimentation rate, Hb: Hemoglobin, Hct: hematocrit, LDL: low-density lipoproteins, PLT: platelets, HDL: high-density lipoproteins, RBC: red blood cells, PCI: percutaneous coronary**,** WBC: white blood cells

Supplemental Table 3. Oxidative stress, endothelial dysfunction and autophagy features.*

Characteristic	TAA Group (N=29)	Control Group (Aortic Valve Surgery) (N=20)	P
**Serum Nox2 - pg/ml**	45.29 (38.07, 55.77)	27.43 (14.17, 40.49)	0.005
**Tissue Nox2**	34.36 (27.23, 40.37)	23.84 (18.11, 29.49)	0.003
**Nox2 serum/tissue ratio**	1.22 (1.15, 1.29)	1.03 (0.76, 1.18)	0.015
**Serum H_2O_2 - µM**	45.61 (37.94, 53.44)	42.53 (22.18, 46.65)	0.020
**Tissue H_2O_2**	51.77 (39.50, 64.31)	39.50 (33.65, 45.15)	0.003
**H_2O_2 serum/tissue ratio**	0.88 (0.83, 0.96)	1.15 (0.64, 1.27)	0.13
**HBA - % of inhibition**	35.42 (13.64, 53.67)	59.35 (43.73, 71.93)	0.003
**Serum NO - µM**	12.49 (7.92, 20.66)	24.72 (17.28, 31.94)	0.001
**Tissue NO**	20.11 (12.50, 32.36)	35.04 (27.29, 42.49)	0.001
**NO serum/tissue ratio**	0.623 (0.605, 0.645)	0.644 (0.633, 0.650)	0.010
**P62 – ng/ml**	95.06 (81.11, 123.67)	70.98 (67.71, 84.46)	<0.001
**ATG5 – ng/ml**	103.10 (85.63, 121.30)	139.93 (105.12, 153.93)	0.003
**ATG5/Actin† – AU**	1.06 (0.55, 1.34)	2.80 (2.12, 5.78)	0.017
**ATG7/Actin† – AU**	0.57 (0.33, 0.72)	1.00 (0.65, 1,55)	0.053
**Beclin/Actin† – AU**	1.18 (0.97, 1.40)	1.44 (1.29, 2.01)	0.21
**LC3/Actin – AU**	2.71 (1.38, 3.34)	8.49 (3.25, 10.40)	<0.001

*presented as median with 25th and 75th percentiles (Q1, Q3) and corresponding p value; †available in 7 subjects in the TAA group and 3 subjects in the control group

AU: arbitrary unit; HBA: hydrogen peroxide breakdown activity; NO: nitric oxide; TAA: thoracic aortic aneurysm

Supplemental Table 4. Correlation analysis between oxidative stress, endothelial dysfunction and autophagy features.*

**Spearman rho, p value**	**Tissue Nox2**	**HBA**	**Serum H2O2**	**Tissue H2O2**	**Serum NO**	**Tissue NO**	**P62**	**ATG5**	**LC3/ Actin**
**Serum Nox2**	**0.761<br><0.001**	0.070<br>0.63	0.221<br>0.13	0.126<br>0.39	**-0.504<br><0.001**	**-0.453<br>0.001**	**0.424<br>0.002**	**-0.408<br>0.004**	-0.205<br>0.16
**Tissue Nox2**	-	-0.143<br>0.33	0.254<br>0.078	0.233<br>0.11	**-0.480<br><0.001**	**-0.407<br>0.004**	**0.380<br>0.007**	**-0.341<br>0.016**	-0.153<br>0.30
**HBA**	-	-	-0.144<br>0.32	**-0.295<br>0.040**	-0.035<br>0.81	-0.132<br>0.37	-0.240<br>0.096	0.117<br>0.42	0.170<br>0.24
**Serum H_2O_2**	-	-	-	**0.650<br><0.001**	0.048<br>0.75	0.010<br>0.95	**0.419<br>0.003**	-0.065<br>0.66	-0.118<br>0.42
**Tissue H_2O_2**	-	-	-	-	0.006<br>0.97	0.043<br>0.77	**0.525<br><0.001**	-0.202<br>0.16	-0.231<br>0.11
**Serum NO**	-	-	-	-	-	**0.944<br><0.001**	**-0.293<br>0.041**	**0.480<br><0.001**	**0.291<br>0.042**
**Tissue NO**	-	-	-	-	-	-	**-0.297<br>0.038**	**0.482<br><0.001**	**0.376<br>0.008**
**P62**	-	-	-	-	-	-	-	**-0.310<br>0.030**	**-0.456<br>0.001**
**ATG5**	-	-	-	-	-	-	-	-	0.276<br>0.055

*nominally significant correlations are highlighted in bold type

Supplemental Table 5. Multivariable linear regression for oxidative stress, endothelial dysfunction and autophagy features.*

**Feature**	**Univariable analysis**	**p value**	**Multivariable analysis excluding aortic dimensions†**	**p value**	**Multivariable analysis including aortic dimensions†**	**p value**
Serum Nox2 - pg/ml	14.4 (4.9, 23.9)	0.004	19.9 (1.3, 38.4)	0.037	25.3 (0.8, 49.7)	0.043
Tissue Nox2	9.0 (3.7, 14.4)	0.001	12.4 (2.2, 22.7)	0.019	16.7 (3.3, 30.1)	0.016
**HBA - % of inhibition**	-21.1 (-34.1, -8.1)	0.002	-17.4 (-40.7, 5.9)	0.14	-13.3 (-44.1, 17.6)	0.47
**H_2O_2 - µM**	10.2 (3.8, 16.6)	0.002	16.2 (5.5, 26.9)	0.004	15.5 (2.8, 28.3)	0.018
Tissue H2O2	14.9 (6.0, 23.9)	0.002	8.8 (-5.5, 23.0)	0.22	8.6 (-10.3, 27.5)	0.36
Serum NO - µM	-10.0 (-15.8, -4.2)	0.001	-10.5 (-19.5, -1.4)	0.024	-15.1 (-26.7, -3.4)	0.013
Tissue NO	-13.1 (-20.9, -5.3)	0.001	-15.5 (-28.5, -2,5)	0.021	-24.3 (-40.8, -7.8)	0.005
**P62 – ng/ml**	22.2 (9.8, 34.7)	0.001	23.3 (0.5, 46.1)	0.045	25.6 (-6.0, 57.1)	0.108
**ATG5 – ng/ml**	-27.7 (-45.0, -10.3)	0.002	-41.2 (-72.4, -9.9)	0.011	-35.4 (-76.7, 5.9)	0.091
**LC3/Actin**	-5.5 (-8.0, -2.9)	<0.001	-3.9 (-7.1, -0.7)	0.017	-6.7 (-12.4, -0.9)	0.025

*using the feature of interest as dependent variable, thoracic aortic aneurysm (coded as 1) vs control (coded as 0) as independent variable alone for the univariable analysis, and also variables associated with group assignment with p<0.05 as per Table 1 (height, body surface area, dyslipidemia), and per Tables 2 and 1S minus aortic dimensions (left ventricular end-diastolic diameter, inter-ventricular septum, severe aortic stenosis, degree of diastolic dysfunction, basophil count, erythrocyte sedimentation rate, and statin therapy), or all the variables above plus maximum aortic diameter, and reported as point estimate of effect (95% confidence interval), and corresponding p values; †significant results were confirmed also using non-linear models
